# Exploring the dynamics of food-related policymaking processes and evidence use in Fiji using systems thinking

**DOI:** 10.1186/s12961-017-0240-6

**Published:** 2017-08-29

**Authors:** Gade Waqa, Marj Moodie, Wendy Snowdon, Catherine Latu, Jeremaia Coriakula, Steven Allender, Colin Bell

**Affiliations:** 1 Pacific Research Center for the Prevention of Obesity and Non-Communicable Diseases (C-POND), College of Medicine Nursing and Health Sciences, Fiji National University, Private Mail Bag, Tamavua, Suva Republic of Fiji; 2 Deakin Health Economics, Centre for Population Health Research, Faculty of Health, Deakin University, Geelong, Australia; 3 Global Obesity Centre, Centre for Population Health Research, Faculty of Health, Deakin University, Geelong, Australia

**Keywords:** Policy implementation, Systems thinking, Evidence use, Policymaking process, Food policymaking

## Abstract

**Background:**

Obesity and non-communicable diseases are significant public health issues globally and particularly in the Pacific. Poor diet is a major contributor to this issue and policy change is a powerful lever to improve food security and diet quality. This study aims to apply systems thinking to identify the causes and consequences of poor evidence use in food-related policymaking in selected government ministries in Fiji and to illicit strategies to strengthen the use of evidence in policymaking.

**Methods:**

The Ministry of Health and Medical Services and the Ministry of Agriculture in Fiji were invited through their respective Permanent Secretaries to participate in the study. Three 180-minute group model building (GMB) workshops were conducted separately in each ministry over three consecutive days with selected policymakers who were instrumental in developing food-related policies designed to prevent non-communicable diseases. The GMB workshops mapped the process of food-related policymaking and the contribution of scientific and local evidence to the process, and identified actions to enhance the use of evidence in policymaking.

**Results:**

An average of 10 policymakers participated from each ministry. The causal loop diagrams produced by each ministry illustrated the causes and consequences of insufficient evidence use in developing food policies or precursors of the specific actions. These included (1) consultation, (2) engagement with stakeholders, (3) access and use of evidence, and (4) delays in policy processes. Participants agreed to potential leverage points on the themes above, addressing pertinent policymaker challenges in precursor control, including political influence, understanding of trade policies, competing government priorities and level of awareness on the problem. Specific actions for strengthening evidence use included training in policy development and research skills, and strengthening of coordination between ministries.

**Conclusions:**

The GMB workshops improved participants’ understanding of how different parts of the policy system interact. The causal loop diagrams and subsequent action plans enabled the identification of systems-level interventions in both ministries to improve evidence-informed policy development. A guide for integrating multi-sectoral consultation and stakeholder engagement in developing cross-cutting policies is currently being developed.

## Background

Pacific Islands have a high burden of non-communicable diseases (NCDs), with some of the highest rates of obesity and diabetes in the world [1–3]. Among them, Fiji is struggling to meet the demands on its national health system and the economic costs of these diseases [4]. The country is starting to look upstream at disease determinants [5, 6], with a view to creating public health policy with the power to influence both individual- and population-level behavioural change [7, 8].

Poor diet is a key determinant of NCDs, and a serious global public health problem [8, 9]. Diets high in fat (saturated fats and trans-fatty acids), free sugars and salt, and low in fruit and vegetable consumption in particular are risk factors for NCDs [10, 11]. The factors involved in dietary choices include income, food prices, individual preferences and beliefs, cultural traditions, and social and economic factors, all of which shape dietary patterns [2, 12, 13]. Structural changes in the food system (shift from locally grown, traditional foods to highly processed imported food) have created an environment where NCD-promoting foods are cheap, abundant and highly palatable. Efforts to reverse these structural changes and improve the healthiness of food environments will need multi-level, multi-actor engagement [7, 14].

Policy is a particularly powerful tool for reshaping food systems since it can influence price, availability and promotion of both healthy and unhealthy foods along the supply chain. With healthier foods available, consumers have more opportunity to make healthier choices [15]. The food systems and food supply chain comprise multiple components, including the growing, harvesting, processing, packaging, transporting, marketing, consuming and disposal of food. Sectors that have the potential to influence the food environment include agriculture, health, trade, manufacturing and marketing, and government [16]; the latter plays a major role in transforming the food supply chain [14, 17, 18].

Food-related policymaking processes vary widely between sectors. We define effective food policies as those that successfully influence any of the key determinants of obesity and poor diet [15]. In order to understand drivers of or obstacles to food-related policymaking, it is essential to understand the underlying systems that generate the dynamic behaviour of a problem over time and to identify issues that are likely to facilitate or hinder the process of policymaking [19–23].

The WHO’s Alliance for Health Policy and Systems has promoted systems thinking as a strategic approach to strengthening health systems in order to achieve global and national health goals [24]. Systems thinking has been used in other disciplines, such as engineering [25], and more recently to improve public health [25–27] and policy systems [26, 28–30]. It provides an approach to problem solving that views problems as part of a wider dynamic system [24, 26, 31, 32].

Drawing on systems thinking, this study aims to identify the causes and consequences of poor evidence use in food-related policymaking in the health and agriculture ministries in Fiji and to illicit strategies to strengthen the use of evidence informed by the following research question: Where could evidence levers be applied within the food-related policymaking processes in Fiji?

## Methods

### Recruitment

A purposive and convenience sampling approach was used to select and recruit policymakers from the Ministry of Health and Medical Services (MoHMS) and the Ministry of Agriculture (MOA). We aimed to recruit senior policymakers who were involved with food-related policymaking, and had the power to influence change in the government system. Both the ministries were chosen because they are key stakeholders in food systems and NCD prevention and policy, and had been previously involved in the Pacific Obesity Prevention In Communities project 2004–2009 [33], and the Translational Research on Obesity Prevention In Communities project 2010–2012 [21, 34].

### Research design

This study used group model building (GMB) and a system dynamics approach [28, 35] to gain insights into the connections between variables influencing the use of evidence in food-related policymaking in the two selected government ministries in Fiji. GMB is a participatory method involving a group of key informants (people with content and knowledge specific to the problem under consideration) in a modelling process that can lead to systems insights and recommendations [35]. It can help in identifying causal relationships, feedback loops, delays and other hindrances to the process of food-related policymaking. One product of the process is a causal loop diagram (CLD), which provides a grounded logic model of the complex drivers of a problem [36, 37] and which can be used to design further strategies to improve policymaking in government organisations. The study used selected food-related policies as case studies to facilitate discussions in the GMB approach, where participants retrospectively reviewed the process employed in the development of these policies and explored their understandings of the system.

### Data collection

We conducted GMB in line with a system dynamics approach [28, 35, 36] to gain insights into the connections between variables influencing evidence use in food-related policymaking in the two selected ministries. Data were collected over three 180-minute GMB workshops over 3 days at a venue away from participants’ usual workplace to help them fully engage in the discussion. The GMB team comprised seven members, namely a team leader/lead facilitator (GW), a co-facilitator (CL), a modeller (JC), three note-takers and a collaborator from Deakin University (Australia) [38]. All team members received practical training in data collection for each step in the process based on a handbook of scripts (Scriptapedia) for developing structured GMB sessions [35, 38]. The data collection processes comprised four sessions spread across the three workshops.

### Session 1: framing a dynamic problem

First, the participants were given an overview of NCDs and the policymaking process to draw on their understanding of the dynamic nature of the underlying drivers. Because of participants’ previous experience in developing policies and consulting and/or engaging with other stakeholders who may be affected by the policy, they were considered to be skilled and interested in addressing dynamic problems in the policy systems. Participants identified causes and consequences of poor evidence use in food-related policymaking within their ministry from the inception of the policy problem through to policy implementation [35, 36]. For each cause and consequence (variable), a graph was developed (change over time graph), comprising time on the horizontal (x-axis) and the variable on the vertical (y-axis) to illustrate how that variable had changed over time and what may happen to it in the future. Participants then shared stories of how the variables influenced evidence use. Two examples of potentially powerful policies in Fiji suggested by participants as case studies using systems approach were ‘Marketing of foods and non-alcoholic beverages to children regulation’ and ‘Integrated Water Resource Management Policy associated with Land Use Policy’. No other policies were suggested by the participants apart from the two case studies mentioned above. Furthermore, participants unanimously agreed that many of the obstacles involved in the selected case studies entailed other sectors; therefore, the case studies allowed participants to retrospectively review the process employed in the development of selected policies and explore their understandings of the system.

### Session 2: connection circles

All variables that participants considered important, feasible and measureable were arranged around a circle on a white board. Participants then identified connections between two or more variables, and specified the direction of causation and the nature of relationships (positive or negative) between them [35, 36]. A positive indicated that variables changed in the same direction while a negative indicated that the relationship was inverse. Researchers validated these connections with participants by discussing each variable name and description and the direction of linkages with them to ensure the right meaning had been captured. Participants were encouraged to add or refine variables so that the problem was fully mapped out.

### Session 3: CLDs

The process of listing and reviewing variables in the connecting circles was also performed simultaneously using Vensim software [39] to create a CLD, whereby participants could visualise connections between variables and identify feedback loops. The CLDs aided the understanding of interrelationships within the system and the cause-effect linkages for the problem.

### Session 4: action plan

After they had time to think about and review the diagrams, participants were invited to a final session where they identified as many action ideas as they could that would improve the use of evidence in food-related policymaking. Note-takers captured discussions and participants placed feedback directly on the CLDs using sticky labels. The action ideas shared by participants in the third and final workshop were collated into four themes; from these, strategies were developed using the World Café approach [38]. Participants self-selected one of four ‘cafes’ and used a systems grid based on the WHO Building Blocks [24] to identify how to embed the action ideas into the policymaking systems of each Ministry [40]. The completed actions and strategies were presented back to the team, where additional ideas were added until agreement was reached on a set of feasible actions likely to make a difference.

### Data management and analysis

Data were analysed after each session. Data collected included change-over-time graphs, connection circles, CLDs, prioritised action ideas, an action plan and the notes on the discussions. The team leader audited all notes for accuracy and to ensure confidentiality.

## Results

### Participants

Eighteen participants from the MoHMS (n = 9) and the MOA (n = 9) participated in separate GMB workshops in August 2015. The majority of participants (72%) were senior managers (such as National Advisors, Directors and Principal level officers) who were directly involved in policymaking, whilst 28% were middle managers (persons directly responsible to the senior managers) with potential to share evidence that influences the policymaking process. The majority (72%) were male. The selection of senior staff ensured a strong understanding of and expertise in policymaking processes, plus awareness of the workings of their organisation. The participants also used relevant and familiar case studies to help understanding of the GMB process.

### CLDs

**Fig. 1:**
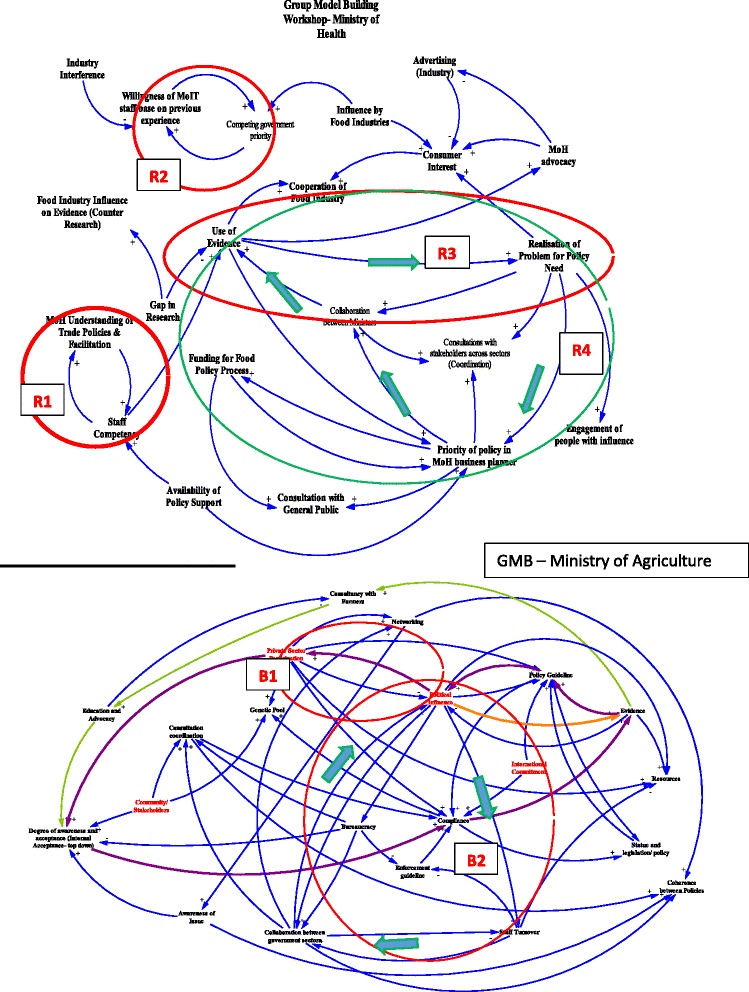
Causal loop diagram showing dynamics of consultation, engagement with stakeholders, access to and use of evidence, delays with all relevant stakeholders policy in business planning, collaboration between ministries, and the use of evidence

The four themes of related variables, connections and feedback loops observed on the CLDs were labelled as (1) consultation; (2) engagement with stakeholders; (3) access and use of evidence; and (4) delays. These dynamics are captured on both maps in Fig. 1.

#### Consultation

Consultation on new policy involves informing and receiving counsel from those with an interest in the policy. It represents the first step in policy development and the aim of consultation is to build trust and start a conversation so that policies reflect individuals’ needs and are relevant to their circumstances.

We identified one balancing feedback loop (B1 or ‘frustration’ loop) and two reinforcing loops (R1 ‘appreciative’ and R2 ‘trust’) [35]. B1 for the MOA demonstrated that increasing political influence from government was balanced by decreasing participation of the private sector. It was named the ‘frustration loop’ because it frustrated MOA policymakers’ efforts to engage with the private sector. The R1 showed that MoHMS staff appreciated some of the training they had on trade policies, which increased their understanding and reinforced their competence and confidence when consulting with trade representatives on the influence of food-related policy on health. R2 demonstrated that trust reinforced cooperation and engagement, and vice versa, such that poor cooperation from the Ministry of Trade on the policy designed to protect the population from the marketing of infant formulas as breast milk substitutes reduced the trust of MoHMS in that Ministry.

#### Engagement with stakeholders

This refers to the willingness of those who have a particular interest in the policy to work together following consultation. Figure 1 also shows a reinforcing (R3 ‘motivation’) loop and a balancing (B2 ‘concern’) loop. R3 demonstrates that increased MoHMS engagement with stakeholders increased the demand for and use of evidence, which in turn increased the realisation of the problem, and therefore the motivation to implement the policy. B2 shows that decreased collaboration within and between government sectors, and with farmers, negatively affected political support and MOA’s operationalisation of the policy within a strict timeline. The absence of adequate collaboration within the organisation has balanced this concern by positively increasing staff turnover in the context of demand for compliance on staff time. Other interactions that contributed to this pathway included staff competency and the priority of including policy in ministry-level business planning. However, there was a growing concern reflected in the balancing loop (B2) (Fig. 1), where decreasing collaboration across government ministries decreased the power of political influence over policy issues, which in turn increased staff turnover, meaning that they either left the ministry or different staff were assigned to the role.

#### Access and use of evidence

This sector of the maps captures factors such as gaps in research that influence access to and use of evidence (Fig. 1). When MoHMS supported the use of evidence in policymaking, this tended to increase the understanding and realisation of the existence of a problem requiring policy intervention (R4 ‘support’ loop). This in turn raised the priority of the proposed policy in the Ministry’s business plan, thereby increasing demand for collaboration between government ministries looping back to an increased demand for evidence.

#### Delays

This theme captures delays in the policy process due to bureaucracy (e.g. demand for compliance of staff time, overwork), politics (e.g. political influence and power) and vested interests (e.g. private sector involvement). This was captured in the concern balancing loop 2 (B2) in Fig. 1, which increased staff turnover in the MOA, and in the reinforcing support loop (R3), which showed the gaps in research that influence success in evidence use. Whilst the MOA staff have analytical and operational skills to work with private sectors, limited information and resources, coupled with multiple roles, limited time and competing deadlines, result in high staff turnover.

### Action plan

The maps were used to help ministry participants identify actions they could be taken to improve evidence use in policymaking. The MOA sought to influence the consultation sector of the map by publishing guidelines on consultation with partners. The MoHMS sought to minimise delays by producing strategic health communication messages to the general population to balance industry’s influence on consumers’ food choices. Both ministries agreed to strengthen engagement by endorsing a government policy for data sharing to improve access and use of evidence. The MoHMS endorsed the development of a guide around multi-sectoral consultation and stakeholder engagement in developing cross-cutting policies, while the MOA opted to build staff capacity in developing policy briefs using reliable evidence.

## Discussion

A GMB process with two government ministries in Fiji revealed that aspects of consultation, engagement with stakeholders, physical access to evidence, and delays due to bureaucracy and vested interests limited the use of evidence in food-related policy development. This study was designed to provide a deeper understanding of the use of evidence by government sectors. However, future GMB processes could include representation from the private sector, which would provide new insights into the CLD. To our knowledge, this is the first time GMB has been used to strengthen food-related policymaking, and outcomes from the process were the development of a consultation guide and training on policy development and research skills. Although the lead researcher had existing relationships with some of the participants, through joint involvement in the Pacific Obesity Prevention In Communities and Translational Research on Obesity Prevention In Communities projects and other research, there were just as many participants without a prior relationship; therefore, we are comfortable that there was no participation bias.

### Consultation

Identifying and consulting with relevant parties is complex, requiring the coordinated efforts of government, private sectors and civil societies [41]. An example of this complexity was when Fiji implemented a ban on mutton flap sales in 2002 under a trading standard regulation; this was not under the mandate of the MoHMS, which contributed to poor enforcement and limited impact [19]. We found that appreciation of other sectors’ work and trust were key ingredients in the consultation phase. For example, taxes on sugar-sweetened beverages have been adopted and then removed multiple times in Fiji due to political priorities, industry and consumer pressure [19, 20, 42]. Our CLD showed a number of interactions with various actors that either affected or were affected by consultation and engagement with relevant stakeholders (Fig. 1). While the MoHMS appreciated having an increased understanding of trade policies (R1), there is a clear lack of enforcement mechanisms in integrating health with trade policy environments [19] and industry and consumer response to taxation remains largely unknown [43]. Trust assumes a key position within the transactional process of information exchange or communication and is developed through repeated personal interactions [44]. Others have identified trust as an important factor for facilitating interactions between actors [45] and in understanding the power of food industries in interfering with the policy process [43]. The action plan addressed appreciation and trust by endorsing a government policy for data sharing to improve access and use of evidence and the guide for multi-sectoral engagement and consultation.

### Engagement with stakeholders

The motivation loop showed in R3 (Fig. 1) is an internal driver that increased awareness of the need for policy and triggered the increased need for collaboration across government sectors, as well as the demand for evidence. It was evident that policymakers realised the importance of strengthening their own professional networks and skills in using evidence when collaborating with partners across sectors. Various strategies were developed to support and motivate senior managers towards increased collaboration with stakeholders, including knowledge translation [21, 46] and policy dialogues [47]. There is a growing interest in collaborative approaches in food policies and the guide will improve and strengthen multi-sectoral engagement between stakeholders. The possibility of including the insights of advisory groups in the policymaking process is another way of improving the use of evidence in policymaking.

### Access and use of evidence

Political and technical support for ensuring data availability and integrity is needed to strengthen the use of evidence in policymaking in Fiji. Policymakers in the health and agriculture ministries were aware of the importance of using evidence in policymaking. However, it is important to note that their initiative of accessing and applying evidence increased understanding of how their organisation’s systems are positioned to support evidence use when developing policies across sectors. The ability to effectively communicate and integrate evidence into the policymaking process requires that policymakers have the knowledge and technical skills to use reliable evidence which adds value and can influence decisions [14, 48]. Other interactions that may affect or are affected by this pathway include the availability of policy support (poor communications and interference of food industries) [15, 49, 50], funding for food policies (the absence of policy in business plans affect funding and prioritisation of a proposed policy) and staff competency [21] in accessing and using evidence. The central focus of this reinforcing support loop (R4) demonstrates a wide and inconsistent range of practices and attitudes towards evidence across government agencies. In fulfilling the government’s responsibilities to a more systematic and whole-of-government approach to the use of evidence in policymaking, an agreement is required to provide policy guidance to sustain commitment across sectors.

### Strengths and limitations of the study

A major strength was the endorsement and high-level support received from selected government Ministries with the sense of ownership that allowed the release of policymakers as participants to the GMB workshops. Scriptapedia helped the team rehearse and choreograph GMB workshops so they ran smoothly.

A limitation was that the total time allocated for workshops was short, especially for action planning, and that there was not enough time to review the CLDs to ensure they reflected the organisation’s views. There was no involvement of other ministries/private sector who contribute to and influence food-related policymaking in Fiji and therefore the views expressed here are limited to those of the health and agriculture ministries. Lastly, the process was new to participants and this may have affected their level of engagement.

## Conclusions

A GMB process, drawing on systems dynamics, helped government ministries in Fiji to identify consultation, stakeholder engagement, access and use of evidence, and delays due to bureaucracy or vested interests as causes and consequences of poor evidence use in the policymaking process, and to develop appropriate remedial actions. In response, a guide for integrating multi-sectoral consultation and stakeholder engagement in developing cross-cutting policies is currently being developed in one ministry.

## Abbreviations

CLD: causal loop diagram
GMB: group model building
MOA: Ministry of Agriculture
MoHMS: Ministry of Health and Medical Services
NCDs: non-communicable diseases
